# Estimation of Engagement in Moderate-to-Vigorous Physical Activity from Direct Observation: A Proposal for School Physical Education

**DOI:** 10.3390/children8020067

**Published:** 2021-01-21

**Authors:** Élvio R. Gouveia, Bruna R. Gouveia, Adilson Marques, Helder Lopes, Ana Rodrigues, Tomás Quintal, Marcelo Pestana, Miguel Peralta, Matthias Kliegel, Andreas Ihle

**Affiliations:** 1 Department of Physical Education and Sport, University of Madeira, 9020-105 Funchal, Portugal; hlopes@uma.pt (H.L.); anajar@staff.uma.pt (A.R.); tomasquintal9@gmail.com (T.Q.); jm.desporto@gmail.com (M.P.); 2 LARSYS, Interactive Technologies Institute, 9020-105 Funchal, Portugal; bgouveia@esesjcluny.pt; 3 Center for the Interdisciplinary Study of Gerontology and Vulnerability, University of Geneva, 1205 Geneva, Switzerland; matthias.kliegel@unige.ch (M.K.); andreas.ihle@unige.ch (A.I.); 4 Saint Joseph of Cluny Higher School of Nursing, 9050-535 Funchal, Portugal; 5 Regional Directorate of Health, Secretary of Health of the Autonomous Region of Madeira, 9004-515 Funchal, Portugal; 6 CIPER, Faculdade de Motricidade Humana, Universidade de Lisboa, Lisbon, 1495-751 Cruz Quebrada, Portugal; amarques@fmh.ulisboa.pt (A.M.); mperalta@fmh.ulisboa.pt (M.P.); 7 ISAMB, Faculdade de Medicina, Universidade de Lisboa, 1649-028 Lisboa, Portugal; 8 Swiss National Centre of Competence in Research LIVES—Overcoming Vulnerability: Life Course Perspectives, Lausanne and Geneva, 1022 Chavannes-près-Renens, Switzerland; 9 Department of Psychology, University of Geneva, 1205 Geneva, Switzerland

**Keywords:** physical education, invasion games, physical activity, observation tool

## Abstract

This study aimed to test an observational momentary time sampling tool to estimate in-class moderate-to-vigorous physical activity (MVPA), in terms of validity, reliability and agreement between observational momentary time sampling and accelerometry, and to develop a regression equation to estimate MVPA from observational momentary time sampling. The sample comprised 78 pupils (38 girls), mean age 14.0 ± 1.1 years. Measurements were taken in three similar Physical Education classes, on three different days. To monitor MVPA, we applied the observational momentary time sampling method. Students wore an ActiGraph GT3X+ accelerometer. Reliabilities were determined by the intraclass correlations, the agreement between methods was analyzed using the Bland–Altman method, and a multiple regression analysis was performed to estimate the equation. The observational momentary time sampling showed good reliability across time (0.59 < *r* < 0.72, *p* < 0.001). It was significantly correlated with accelerometry (*r* = 0.51, *p* < 0.001). The MVPA assessed via accelerometer could be predicted from the following equation: Y = 44.3 + 0.47⋅(MVPA observational momentary time sampling method) + 8.0⋅(sex; with 0 = girls and 1 = boys). This observational momentary time sampling method is a stable and reliable tool to estimate MVPA. A regression equation using the score of observational momentary time sampling and sex can be used to better estimate the real MVPA.

## 1. Introduction

Exercising moderate-to-vigorous physical activity (MVPA) provides health benefits for children and youth [1,2]. In particular, an appropriate weekly frequency, intensity, and time of physical activity (PA) contributes to the development of strong musculoskeletal tissues [3], efficient cardiovascular system [4], neuromuscular awareness [5], and facilitates the maintenance of a healthy body composition [6]. Moreover, significant psychological benefits [7] and cognitive improvements [8] have also been found as a result of increased daily MVPA.

Currently, for children and adolescents who are less active in their leisure time, Physical Education classes at school represent the only regular opportunity to engage in structured PA [9]. For this reason, it has been argued that schools play an important role in PA promotion [10,11]. However, the recent literature indicates that PA levels in Spanish children and adolescents during schools’ Physical Education classes are often very low [12]. Therefore, the students’ time exercising MVPA spent in-class is considered an important indicator to assess the quality of Physical Education lessons in terms of engagement [13,14,15]. It has been suggested that children and adolescents should spend at least 50% of their time in Physical Education classes on MVPA. A recent systematic review and meta-analysis on MVPA levels in Physical Education lessons [10] showed that 60% of the studies concluded that Physical Education classes fail to achieve those crucial recommendations. This underlines the importance of objectively monitoring the MVPA levels in Physical Education classes, as a basis to reduce the time spent on class management (i.e., instruction and organization). As consequence, an improvement in the global quality and benefits of the Physical Education classes will be achieved [14]. 

Only a few reliable and valid paper-and-pencil worksheets to estimate MVPA in physical education classes are available. In their systematic review and meta-analysis, Hollis et al. [10] showed that twelve studies used observational measures ((SOFIT) [16] and Simple Activity Measurement (SAM) [17]), seven used accelerometers, five used heart rate monitors, and four used pedometers to monitor MVPA. The assessment of in-class PA is difficult for Physical Education teachers, since most of the available tools and technologies are neither economical nor effective for appropriately measuring MVPA in the school context. For example, the most objective measures, such as accelerometers, global positioning sensors (GPS), and heart-rate monitors, are expensive and cause significant difficulties for daily application in all Physical Education activities. On the other hand, although the most frequently used observational methods, such as SOFIT [16] and SAM [17], are economical, reliable, and valid to assess in-class PA, they can be too complex and time-consuming for everyday use. In addition, in daily practice, those instruments seem to be inadequate for use by students in peer- or self-evaluation.

Considering the issues on feasibility of observational methods to estimate the MVPA in-class, Siedentop, Hastie, and Mars [18] have proposed a simple paper-and-pencil worksheet to estimate in-class MVPA, using an observational approach called momentary time sampling. This instrument was presented in the context of the Sport Educational Model, where students have different roles in classes, including observers [18]. The use of this instrument consists of selecting three students and, every 120 s, observing whether they are engaged in MVPA. At the end of an observation cycle, it is possible to estimate the overall time spent in in-class MVPA (as a percentage).

To the best of our knowledge, to date, an evaluation of the validity and reliability of this brief, economical, simple, and practical tool to monitor PA in-class is missing. Therefore, the present study set out to: (1) evaluate the stability (reliability) of the observational momentary time sampling tool to assess MVPA and the agreement in relation to MVPA measurement via accelerometry, and (2) to develop a regression equation to calibrate the score of MVPA% obtained by the observational momentary time sampling tool, to be used in the invasion games on Physical Education context.

## 2. Materials and Methods 

### 2.1. Participants

The sample comprised 78 pupils (40 boys and 38 girls), mean age 14.0 years (SD = 1.1, 95% CI: 13.8–14.2), mean body mass 57.4 kg (SD = 1.8; 95% CI:53.9–61.0), mean height 160.4 cm (SD = 1.0; CI 95% 158.3–162.4), from the 7th to the 9th year, who participated in the 2019 research project entitled “Physical Education in Schools from the Autonomous Region of Madeira” in Funchal, Portugal. This study was carried out in three classes of invasion games in the Physical Education context, in two urban public elementary and secondary schools from the city of Funchal, Madeira, Portugal. A similar lesson plan, using the tactical games approach, was followed over the course of three Physical Education classes. Following this pedagogical approach, the instructional unit enables students to learn and to address similar tactical problems across different invasion games, such as soccer, handball and basketball [19]. Teaching and training progressions are based on small-sized games (two vs. two players, three vs. three players), progressing to a maximum of six players per team on the advanced level [20]. 

Participants were informed about the objectives of the study and written informed consent was obtained from their legal guardians. The study received ethical approval from the Scientific Committee of the Faculty of Physical Education and Sports at the University of Madeira (Reference: ACTA N.77-12.04.2016). This study was also approved by the Regional Secretary of Education and the school’s headmaster. The study was conducted in accordance with ethical standards in sports exercise research [21]. 

### 2.2. Measures

#### 2.2.1. Moderate-to-Vigorous Physical Activity from Observational Momentary Time Sampling Tool

MVPA was estimated during 60-min Physical Education lessons of invasion games on three different days over 3 weeks. These assessments occurred in the same weekday, in parallel, for each class. For this purpose, a paper-and-pencil worksheet was used, and observation was done following an observational momentary time sampling approach, as suggested by Siedentop et al. [18]. Each researcher selected three students, and every 120 s, a scan was done in order to identify whether they engaged in MVPA. With this quick snapshot of what students were doing, a yes-or-no decision was made. A student lying down, sitting, or standing would be identified as sedentary and classified with “no”. If students were engaged in activities that required energy expenditure of at least brisk walking, running, or jumping, they would be classified as engaged in MVPA (i.e., a “yes” would be recorded). A detailed description of the evaluation procedures, namely, protocols, movements classification and scoring, can be found in the Complete Guide to Sport Education 2nd edition [18]. During the 60-min lesson with observation intervals of 120 s, 15 observations were collected for each student. In total, 45 observations were collected for each student across the three different days over three weeks. The assessments were performed by trained researchers. To maximize the consistency of the assessment procedures, three training sessions were conducted. First, a theoretical explanation of the protocol was provided to all field-team members. Second, training sessions were conducted in a class with similar characteristics. Finally, a discussion session with all field-team members was promoted in order to finalize standardization of procedures to maximize accuracy in all observations.

#### 2.2.2. Moderate-to-Vigorous Physical Activity from Accelerometry

During the same 60-min Physical Education lessons, MVPA was also tracked using ActiGraph GT3X+ accelerometers, the most objective and frequent devices used by researchers, accounted for in more than half of the published studies [22]. Students were asked to wear the accelerometer on their right hip. The ActiGraph GT3X+ accelerometer was initialized with a 30 Hz sampling frequency and raw data from gt3x files were converted to 10-s epoch data files prior to analysis. Time spent in MVPA were derived using the ActiLife software, version 6 (ActiGraph, Pensacola, FL, USA), using the cutoff points suggested by Freedson, Melanson, and Sirard [23]. The accelerometer was programmed before each lesson and the data collection started 10 min after the beginning of the lesson. At the same time, the assessments with the observation tool were started.

### 2.3. Data Analysis

First, all data were tested for normality (i.e., Kolmogorov–Smirnov test) and preliminary analyses were performed to ensure no violation of the assumptions (i.e., homogeneity of variances). Second, descriptive statistics for MVPA were calculated separately for boys, girls, and the total sample. An independent-samples *t*-test was conducted to compare the MVPA percentages of each day between boys and girls. Third, the intraclass correlations (ICC) and 95% confidence intervals were calculated to inspect the reliability between repeated measures with the same instrument. We also inspected the correlation between the MVPA measurement via accelerometry and MVPA measurement via observational momentary time sampling methods using the Pearson product–moment correlation coefficient. Fourth, we analyzed the agreement between methods using the Bland and Altman method [24]. Finally, to estimate the regression equation of MVPA measurement via accelerometry, we performed a multiple regression analysis. Different predictors, such as age, MVPA measurement via observational momentary time sampling and sex were tested. However since, age was not a significant predictor, the final model only included the estimation of MVPA measurement via observational momentary time sampling and sex as main predictors. Heteroscedasticity was tested using the Breusch–Pagan test. Data analysis was performed using IBM SPSS v24 (IBM Corp., Armonk, NY, USA). The significance level was set at *p* < 0.05.

## 3. Results

### 3.1. Descriptive Statistics

Means and standard deviations in boys, girls, and for the total sample of MVPA assessed via accelerometer, and observational momentary time samplings are displayed in Table 1. An independent-samples *t*-test was conducted to compare the MVPA percentages of each day for boys and girls. There was only a significant difference in the MVPA assessed via accelerometer in day 2 and day 3, with boys showing higher levels. No other significant difference was seen.

### 3.2. Stability of the Measures between MVPA Measurement via Observational Momentary Time Sampling and MVPA Measurement via Accelerometry Methods

Good reliability was observed for the MVPA measurement via accelerometry and MVPA measurement via observational momentary time sampling methods between day 1 and day 2, day 1 and day 3, and day 2 and day 3 (Table 2). The MVPA measurement via accelerometry reliability ranged between 0.69 (*p* < 0.001) and 0.87 (*p* < 0.001), slightly higher than for MVPA measurement via observational momentary time sampling that ranged between 0.59 (*p* < 0.001) and 0.72 (*p* < 0.001). The correlations between MVPA measurement via accelerometry and MVPA measurement via observational momentary time sampling were: Day 1 vs. Day 1, *r* = 0.30, *p* = 0.021; Day 2 vs. Day 2, *r* = 0.60, *p* < 0.001; Day 3 vs. Day 3, *r* = 0.40, *p* = 0.003, and collapsed across the three days *r* = 0.51, *p* < 0.001. 

### 3.3. Agreement between MVPA-Observational Momentary Time Sampling and MVPA Measurement via Accelerometry Methods

Table 3 shows the mean of paired data (collapsed across the three days), from which the Bland–Altman analyses was constructed to evaluate the agreement between assessment methods. The average differences between MVPA-observational momentary time sampling and MVPA measurement via accelerometry methods is -33.4%. This means that, on average, the MVPA measurement via accelerometry method measures 33.4% in terms of MVPA%, more than the MVPA-observational momentary time sampling method. The average of the differences is normally distributed (assessed by Kolmogorov–Smirnov test). Then, the standard deviation was used to define the limits of agreement. Standard deviation = 12.54, and Mean 33.36 were used to calculate that the 95% Confidence Interval will be: Lower bound= 33.36−(12.54⋅1.96) = 8.78; Upper Bound = (12.54⋅1.96) + 33.36= 57.94

**Figure 1:**
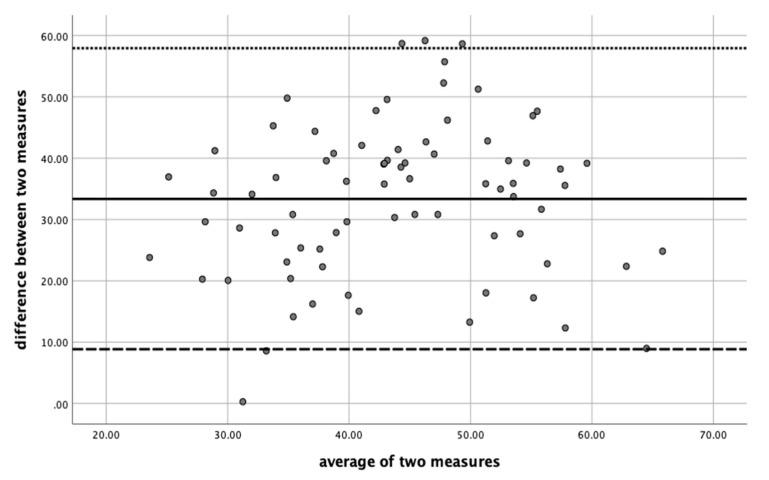
Bland–Altman plot of agreement between MVPA-observational momentary time sampling and MVPA measurement via accelerometry.

The results measured by MVPA-observational momentary time sampling could range from 8 to 58 units of MVPA percentage below the MVPA measurement via accelerometry. In other words, the percentage of MVPA was significantly underestimated by the MVPA observational momentary time sampling method. The Bland–Altman plot of agreement between methods are presented in Figure 1. 

### 3.4. Regression Equation Using the MVPA Measurement via Observational Momentary Time Sampling and Sex to Estimate the MVPA Measurement via Accelerometry In-Class

Finally, to estimate the MVPA assessed via accelerometer in-class, a multiple regression analysis was performed, with the estimation of MVPA assessed via observational momentary time sampling (in percentage) and sex (0 = girls and 1 = boys) as main predictors. The Breusch–Pagan test identified no Heteroscedasticity (*p* = 0.839). The following regression equation should be used to calibrate the MVPA percentage provided by observational momentary time sampling Y = 44.3 + 0.47⋅(X) + 8.0⋅(sex), where X = percentage of time in MVPA assessed by observational momentary time sampling. VIF = 1.59; R^2^ = 0.37; SEE = 10.7; Durbin-Watson = 1.50. Observational momentary time sampling score (*B* = 0.48; *p* < 0.001) and sex *(B* = 0.34; *p* = 0.002) were significant predictors.

## 4. Discussion

This study aimed to evaluate the validity of a brief, economical, simple and practical tool to estimate the real MVPA in Physical Education classes. The paper-and-pencil worksheet presented by Siedentop et al. [18] was found to be a reliable tool in classes of invasion games. In addition, there was a moderate, positive correlation between the assessment of MVPA by the accelerometer and the estimation of MVPA by observational momentary time sampling. However, the MVPA measurement via accelerometry method measures 33.4% more than the MVPA measurement via the observational momentary time sampling method. This means that the percentage of MVPA in-class was underestimated when using the MVPA-measurement via the observational momentary time sampling method alone. Consequently, for calibration purposes, a regression equation using the MVPA-measurement via observational momentary time sampling score and sex was developed to estimate the percentage time spent in MVPA, assessed via accelerometer. To the best of our knowledge, this is the first study that evaluates the validity of this observational tool using ActiGraph GT3X+ as a criterion measure.

First, the reliability of MVPA measurement via observational momentary time sampling was similar to MVPA measurement via the accelerometry method, which is considered a gold standard for the assessment of PA in Physical Education classes [22]. In both methods, the reliability was of a good size, which means that those instruments were stable over time, over the course of three assessments. The reliability results for our observational momentary time sampling tool are comparable to other observational measures, such as SAM (R = 0.83) [17] and SOFIT (R ≥ 0.75) [16,25,26,27]. The present data support the idea that the MVPA estimated by observational momentary time sampling can be reliably used for the estimation of MVPA in invasion games classes, in the school context. In addition, we observed a substantial correlation of the MVPA estimate by observational momentary time sampling tool and MVPA assessed by accelerometer, indicating a moderate, positive correlation between two variables (*r* = 0.51, *p* < 0.001) with a high MVPA estimate from observational momentary time sampling associated with a higher percentage of MVPA, assessed with the accelerometer.

Second, the agreement between the two measurements was evaluated. The differences between MVPA-measurement via observational momentary time sampling method and MVPA measurement via accelerometry (the reference method) were studied. Our results show that, on average, the MVPA measurement via accelerometry method measures 33.4% in terms of MVPA more than the MVPA-measurement via observational momentary time sampling method, and the limits of agreement may range from 8 to 58 units below. This difference is substantial and should not be ignored when the MVPA-measurement via the observational momentary time sampling method is used alone. This study shows that the percentage of MVPA measurement via observational momentary time sampling is underestimated, taking the MVPA measurement via accelerometry method into consideration. 

Finally, addressing this last finding, to provide a calibration, a regression equation was developed using the percentage of MVPA calculated from the observational tool and sex to estimate the MVPA percentage provided by the ActiGraph GT3X+. The linear regression revealed that sex and the score of MVPA assessed by the observational tool were significant predictors. We can conclude that this equation should be considered by the Physical Education teachers in order to better estimate the students’ time spent in MVPA when they are using the paper-and-pencil worksheet presented by Siedentop et al. [18].

### Limitations and Strengths

We acknowledge some limitations of the present study. First, the small sample size and reduced observations. In this study, the optimal sample size was calculated for Correlation: Bivariate normal model, using G*Power3 [28]. A priori, bivariate correlation indicated that a total sample size of 83 was needed to achieve 95% power to detect a correlation (pH1) of 0.35 at the 0.05 level of significance. Then, the reliability and correlation coefficients between methods were slightly lower than those reported for other observational instruments, such as SAM and SOFIT. Therefore, increasing the number of observations and the sample of students in each observed cycle could improve the reliability and the strength of the correlations. Second, this study was carried out only in invasion games’ Physical Education lessons. Therefore, its validity in other physical activities needs confirmation.

Important strengths are also found in the present study, namely, the thorough evaluation of a brief, economical, simple and practical MVPA observational tool, which could be useful to address the engagement in MVPA in invasion games classes. Due to its features, this instrument is affordable, simple, and time-efficient for school use, when compared to other MVPA measurement tools (e.g., accelerometers, heart rate monitors, pedometers, and other observational tools such as SOFIT and SAM). In addition, in daily practice, this tool seems to be acceptable to be used by students in peer or retrospective self-evaluation using videos of the activities. In this regard, to provide a practical recommendation for the number of direct observations required, based on our investigations, we suggest that an observation each 120 s, or at least 15 observations in total per physical education class session, is suitable. Future research might focus on the generalization of this observational instrument to other type of physical activities. 

## 5. Conclusions

In summary, the MVPA measurement via the observational momentary time sampling method is a stable and reliable tool to estimate MVPA in invasion games classes. However, it should be noted that the percentage of MVPA in class is underestimated, when using the MVPA measurement via the observational momentary time sampling method alone. This study proposes a regression equation, using the estimation of MVPA percent from observational momentary time sampling and sex, to better measure the in-class MVPA. Further evaluation of the validity and reliability of this equation, in larger samples and over a variety of physical activities, is necessary.

## Figures and Tables

**Table 1:** Descriptive statistics of MVPA assessed by accelerometer and direct observation in boys and girls by day (%).

Assessments	MVPA Accelerometer			MVPA Direct Observation		
	Boys	Girls	Total	Boys	Girls	Total
		Mean (*SD*)			Mean (*SD*)	
Day 1	58.2 (22.1)	48.7 (20.3)	53.6 (21.7)	24.0 (15.3)	23.6 (18.8)	23.8 (16.9)
Day 2	68.1 (11.7)	55.6 (15.6) ***	62.1 (15.0)	26.5 (13.2)	28.2 (21.1)	27.3 (17.3)
Day 3	67.5 (8.5)	55.2 (12.1) ***	61.5 (12.0)	24.0 (16.8)	17.3 (18.1)	20.8 (17.7)
Mean	65.1 (12.0)	54.5 (13.6) ***	60.0 (13.8)	25.0 (11.5)	24.1 (16.1)	25.0 (13.7)

*** *p* < 0.001; *SD,* standard deviation; MVPA, Moderate to Vigorous Physical Activity.

**Table 2:** Intraclass correlation coefficients between the repeated assessments performed on the three days, for accelerometry and direct observation.

Assessments Days	Accelerometry ICC (95% CI)	Direct Observation ICC (95% CI)
Day 1 vs. Day 2	0.75 (0.59–0.84) ***	0.63 (0.38–0.76) ***
Day 1 vs. Day 3	0.69 (0.49–0.81) ***	0.59 (0.33–0.75) ***
Day 2 vs. Day 3	0.87 (0.78–0.92) ***	0.72 (0.54–0.83) ***

*** p < 0.001; R, reliability; ICC, intraclass correlations; CI, confidence interval.

**Table 3:** Hypothetical data for an agreement between MVPA-measurement via observational momentary time sampling and MVPA measurement via accelerometry to assess the percent of MVPA.

	MVPA-OTS (A)	MVPA-AC (B)	Mean (A + B)/2	(A−B)	(A−B)/ Mean (%)
Mean	27.22	60.58	44.00	−33.36	−78.84
Standard deviation	11.05	12.21	9.81	12.54	31.85
Maximum	60.00	79.32	65.82	−0.28	−0.89
Minimum	6.70	31.39	23.57	−59.17	−146.86
CI (95%)	24.71–29.72	57.80–63.35	41.67–46.12	−36.20–30.51	−86.07–71.61

CI, confidence interval; MVPA-OTS, Moderate to Vigorous Physical Activity assessed via observational momentary time sampling; MVPA-AC, Moderate to Vigorous Physical Activity assessed via accelerometry.

## Data Availability

The data presented in this study are available on request from the corresponding author.

## References

[1] Janssen I., Leblanc A.G. (2010). Systematic review of the health benefits of physical activity and fitness in school-aged children and youth. Int. J. Behav. Nutr. Phys. Act..

[2] Piercy K.L., Troiano R.P., Ballard R.M., Carlson S.A., Fulton J.E., Galuska R.D. (2018). The Physical Activity Guidelines for Americans. J. Am. Med. Assoc..

[3] Aronson E.A. (2016). NAON Position Statement: Promoting Musculoskeletal Health through Physical Activity for All Children and Adolescents. Orthop. Nurs..

[4] Cristi-Montero C., Chillón P., Labayen I., Casajus J.A., Gonzalez-Gross M., Vanhelst HELENA Study Group (2019). Cardiometabolic risk through an integrative classification combining physical activity and sedentary behavior in European adolescents: HELENA study. J. Sport Health Sci..

[5] Faigenbaum A.D., Farrell A., Fabiano M., Radler T., Naclerio F., Ratamess N.A., Myer G.D. (2011). Effects of integrative neuromuscular training on fitness performance in children. Pediatric Exerc. Sci..

[6] Ortega F.B., Ruiz J.R., Castillo M.J. (2013). Physical activity, physical fitness, and overweight in children and adolescents: Evidence from epidemiologic studies. Endocrinol. Nutr. Órgano Soc. Española Endocrinol. Nutr..

[7] Floriani V., Kennedy C. (2008). Promotion of physical activity in children. Curr. Opin. Pediatrics.

[8] Bidzan-Bluma I., Lipowska M. (2018). Physical Activity and Cognitive Functioning of Children: A Systematic Review. Int. J. Environ. Res. Public Health.

[9] Frömel K., Svozil Z., Chmelík F., Jakubec L., Groffik D. (2016). The Role of Physical Education Lessons and Recesses in School Lifestyle of Adolescents. J. Sch. Health.

[10] Hollis J.L., Sutherland R., Williams A.J., Campbell E., Nathan N., Wolfenden L., Wiggers J. (2017). A systematic review and meta-analysis of moderate-to-vigorous physical activity levels in secondary school physical education lessons. Int. J. Behav. Nutr. Phys. Act..

[11] Pate R.R., Davis M.G., Robinson T.N., Stone E.J., McKenzie T.L., Young J.C. (2006). Promoting Physical activity in children and youth: A leadership role for schools: A scientific statement from the American Heart Association Council on nutrition, physical activity, and metabolism (physical activity committee) in collaboration with the councils on cardiovascular disease in the young and cardiovascular nursing. Circulation.

[12] Grao-Cruces A., Segura-Jiménez V., Conde-Caveda J., García-Cervantes L., Martínez-Gómez D., Keating X.D., Castro-Piñero J. (2019). The Role of School in Helping Children and Adolescents Reach the Physical Activity. Recommendations: The UP&DOWN Study. J. Sch. Health.

[13] Harris J. (2015). Association for Physical Education Health Position Paper. https://dspace.lboro.ac.uk/2134/24152.

[14] Kim M., Jung J. (2019). Application of Instructional Alignment to Promote Moderate-to-Vigorous Physical Activity during Physical Education. Strategies.

[15] U.S. Department of Health and Human Services, Centers for Disease Control and Prevention, National Center for Chronic Disease Prevention and Health Promotion, Division of Adolescent and School Health (2010). Strategies to Improve the Quality of Physical Education. https://www.cdc.gov/healthyschools/pecat/quality_pe.pdf.

[16] McKenzie T.L., Sallis J.F., Nader P.R. (1992). SOFIT: System for Observing Fitness Instruction Time. J. Teach. Phys. Educ..

[17] Surapiboonchai K., Furney S.R., Reardon R.F., Eldridge J., Murray T.D. (2012). SAM: A tool for Measurement of Moderate to Vigorous Physical Activity (MVPA) in school physical education. Int. J. Exerc. Sci..

[18] Siedentop D., Hastie P.A., Van der Mars H. (2011). Complete Guide to Sport Education.

[19] Mitchel S.A., Oslin L.J., Griffin L.L. (2013). Teaching Sport Concepts and Skills: A Tactical Games Approach for Ages 7 to 18.

[20] Gouveia E.R., Gouveia B.R., Marques A., Kliegel M., Rodrigues A.J., Prudente P., Lopes H., Ihle A. (2019). The Effectiveness of a Tacti-cal Games Approach in the Teaching of Invasion Games. J. Phys. Educ. Sport.

[21] Harriss D.J., Atkinson G. (2011). Update—Ethical standards in sport and exercise science research. Int. J. Sports Exerc. Med..

[22] Wijndaele K., Westgate K., Stephens S.K., Blair S.N., Bull F.C., Chastin S.F., Healy G.N. (2015). Utilization and harmonization of adult accelerometry data. Med. Sci. Sports Exerc..

[23] Freedson P.S., Melanson E., Sirard J.R. (1998). Calibration of the computer science and applications, Inc. accelerometer. Med. Sci. Sports Exerc..

[24] Giavarina D. (2015). Understanding Bland Altman analysis. Biochem. Med..

[25] McKenzie T.L., Kahan D. (2008). Physical activity, public health, and elementary schools. Elem. Sch. J..

[26] McKenzie T.L. (2010). 2009 McCloy Lecture Seeing Is Believing: Observing Physical Activity and Its Contexts. Res. Q. Exerc. Sport.

[27] Sharma S., Chuang R.J., Skala K., Atteberry H. (2011). Measuring physical activity in preschoolers: Reliability and validity of The System for Observing Fitness Instruction Time for Preschoolers (SOFIT-P). Meas. Phys. Educ. Exerc. Sci..

[28] Faul F., Erdfelder E., Lang A., Buchner A. (2007). G*Power 3: A flexible statistical power analysis program for the social, behavioral, and biomedical sciences. Behav. Res. Methods.

